# Editorial: The Parallel March of Asthma and Allergy in Childhood: A Multi-Perspective Approach

**DOI:** 10.3389/fped.2018.00135

**Published:** 2018-05-09

**Authors:** Carlo Caffarelli, Kostas Priftis, Carla Mastrorilli, Luis Garcia-Marcos

**Affiliations:** ^1^Clinica Pediatrica Unit, Department of Medicine and Surgery, University of Parma, Parma, Italy; ^2^Pediatric Allergy and Pulmonology Units, Third Department of Pediatrics, School of Medicine, University General Hospital Attikon, National and Kapodistrian University of Athens, Athens, Greece; ^3^Pediatric Allergy and Pulmonology Units, Hospital Universitario Virgen de la Arrixaca, IMIB-Arrixaca Bio-health Research Institute, University of Murcia, Murcia, Spain

**Keywords:** allergy, allergic rhinitis, asthma, atopy, children, exercise-induced bronchospasm, food allergy, united airway disease

Asthma is often associated with atopy and it can be elicited by allergens in some individuals. Frontiers in Pediatrics has dedicated a series of article on asthma and atopy. This research topic aimed to summarize current knowledge and assess various aspects that are useful for improving our understanding.

Asthma, eczema, allergic rhinitis, and food allergy are typically considered as allergic diseases. The sequence of disease progression in childhood is often referred to as the “atopic march.” The connection existing between upper and lower respiratory tract allergic diseases, such as asthma and allergic rhinitis (AR), has been called United Airway Disease (UAD) (Licari et al.). Asthma and AR can be considered manifestations of a single inflammatory process. They share macroscopic pathological characteristics, similar histological appearance, with a comparable allergic response and coexisting symptoms, including cough. The interaction between nose and lung in allergic airways disease is a bidirectional process, indeed it has been proved that the treatment of AR can improve asthma symptoms (Licari et al.). Overall, the causal relationship between atopic diseases in the “atopic march” is still unclear as it is not always present in all patients and the timeline may vary. Genetics and environmental exposure have been shown important risk factors for developing asthma and atopy in childhood, probably through epigenetic mechanisms. However, gene-environment interactions have been rarely examined for both asthma and atopy in the same population and there is no consistent evidence that identical interactions are shared to asthma and atopy (Turner). On the other hand, a defect in regulatory T cells (Tregs) at birth predisposes to atopy and asthma occurrence by enhancing release of Th2 cytokines in response to allergens (Martín-Orozco et al.). Furthermore, allergen-specific Tregs reduce inflammation and remodeling in the airways (Martín-Orozco et al.).

## Risk factors

Asthma has been recognized to be a syndrome since it likely comprehends different conditions. Efforts have been going for a long time to classify asthma according to causes and clinical features (phenotypes) or patophysiological mechanisms (endotypes) (Guibas et al.). The early-onset asthma phenotype is strongly associated with atopy. It starts in childhood and adolescence and it is mainly Th-2 driven (Th2 endotype). Th2 endotype is characterized by atopy, elevated IgE, and airway eosinophilic inflammation. The early-onset asthma phenotype includes atopic asthma which represents the most common form in childhood and it is characterized by positive IgE tests for the relevant allergens whose exposure induces asthma exacerbations (Comberiati et al.). Subphenotypes of early-onset asthma include transient wheeze due to viral infections associated with no eosinophilic inflammation and viral-induced asthma. Atopic status has an influence on such variants (Guibas et al.). Children with elevated concentrations of specific IgE are at higher risk for asthma attacks provoked by viral infections (Comberiati et al.). Viral infections promote allergic airway inflammation that results in asthma attack. Thus, sensitization to aeroallergen and respiratory infections synergistically enhance the risk for asthma. In children, other phenotypes (Guibas et al.) are persistent wheeze (non-eosinophilic asthma endotype) and obesity-induced asthma characterized by a no-/low-Th2 endotype and non-atopic, that should be distinguished from decrease in FEV1/FVC due to overweight by increased airway resistance (Jones et al.). Atopy is also a main risk factor for exercise-Induced bronchospasm (EIB). Up to 40% of children with EIB have AR and 30% of these children have asthma (Caggiano et al.). Identification of allergens associated with EIB permits to adopt measures to reduce allergen exposure. This and administration of the most proper pre-medication will allow children with EIB to perform physical activity as others do.

Considering the role of allergy in asthma occurrence, it is not surprising that atopic sensitization, especially or to dog, cat, any of perennial allergens or concurrent sensitization to airborne allergens and food allergens is linked to higher risk for development of asthma in young children (Moustaki et al.). Accordingly, several score for predicting asthma occurrence have included atopic features in combination with other factors with positive results even if they are rarely used in clinical practice (Moustaki et al.).

Regarding food allergy, it is noteworthy that food allergic children are not only at increased risk for asthma onset and food-induced asthmatic episodes, but also for more severe asthma (Foong et al.).

Another link between asthma and atopy is that emerging evidences indicate that maternal prenatal stress can result in development of both atopy and asthma (Douros et al.).

## Prevention

Avoidance of airborne or food allergens both by mothers in pregnancy and during lactation and by infants does not seem to be of benefit for primary prevention of childhood asthma (Moustaki et al.). In children with AR due to grass pollen, both types of allergen immunotherapy (AIT), subcutaneous (SCIT) and sublingual immunotherapy (SLIT) are effective in preventing onset of asthma (Tsabouri et al.). It has been proposed that asthma occurrence in offspring depends on maternal vitamin D status in pregnancy. However, prenatal levels of Vitamin D are inconsistently associated with onset of asthma in offspring. In randomized controlled clinical trials, supplementation with vitamin D in pregnant women does not reduce the risk of having asthma or whezing up to 3 years of age child (Bountouvi et al.).

A systematic review of observational studies (Castro-Rodriguez and Garcia-Marcos) on the preventive effect of Mediterranean Diet (MedDiet) consumption on atopic diseases found that in children MedDiet seems to prevent asthma/wheezing but not atopy, AR and atopic eczema. Moreover, MedDiet by the mother during pregnancy revealed some protective effect on asthma/wheeze symptoms in the offspring only up to 1 year of age. MedDiet may be of benefit because it is rich in antioxidant (Jesenak et al.). Randomized control trials on the effect of MedDiet are compulsory.

Recently, occurrence of atopy and asthma have been linked to changes in the microbiome. However, there is low evidence that administration of probiotics during pregnancy and in the newborn may prevent atopic eczema, while probiotics are not useful for the primary prevention of childhood asthma (Mennini et al.).

## Treatment

In allergic asthma triggered by the exposure to indoor allergens, allergen avoidance may be a specific preventive measure (Cipriani et al.). Several means may help to reduce exposure to allergens, such as house dust mite, pets, cockroach, molds. Notwithstanding evidence supporting the efficacy of these measures is weak and subject of controversy, the exposure control to specific airborne allergens is widely recommended.

Oxidative stress plays a pathogenetic role in asthma. Asthma is inconsistently associated with low levels of various antioxidants. Contrasting results have been shown by studies on the effect of antioxidant supplementation on asthma (Guibas et al.).

There is a need of improving control of severe asthma in children since it is responsible of morbidity and use of about 50% of health-care funds (Martin Alonso et al.). Serious and multiple allergies, steroid-resistant eosinophilia, and airway remodeling go with severe asthma. Nowadays, the only add-on therapy licensed for use in children with severe asthma is omalizumab. Nevertheless, its use is limited in one-third of patients by higher serum IgE levels than recommended and in another one-third by clinical weakness. Therefore, pediatric severe asthma is heterogeneous of, and mechanisms underlying different phenotypes are still unknown. Two add-on treatments, monoclonal antibodies to IL-5 or its receptor, and CRTH2 antagonists, currently available for use in adults, can be attractive options for children with severe asthma. They may help to improve the persistent steroid resistant eosinophilia.

AIT appears to be effective in children with IgE-mediated asthma who do not fully respond to the conventional anti-asthmatic medications and environmental control (Tsabouri et al.). Both SCIT and SLIT reduce asthma symptoms and medication use. It is important to underline that AIT is the only treatment that may change the natural history of respiratory allergy. Severe, not controlled asthma, and medical inaccuracy were the most frequent causes of SCIT-induced adverse event. In the era of personalized medicine, further research should be conducted to individualize AIT using recombinant antigen technology. Allergen extracts against specific proteins to which the patient is allergic or extracts with modified proteins or peptides that could increase safety/efficacy might be created.

## Author contributions

CC designed and wrote the article. CM wrote the article. KP and LG-M equally designed the article, read, and made comments on the manuscript.

### Conflict of interest statement

The authors declare that the research was conducted in the absence of any commercial or financial relationships that could be construed as a potential conflict of interest.

